# Supplementary figures and images for: High-Dose Opioid Prescribing and Opioid-Related Hospitalization: A Population-Based Study
Source: PLoS One. 2016 Dec 14;11(12):e0167479. doi: 10.1371/journal.pone.0167479 (PMC5156349; doi:10.1371/journal.pone.0167479)

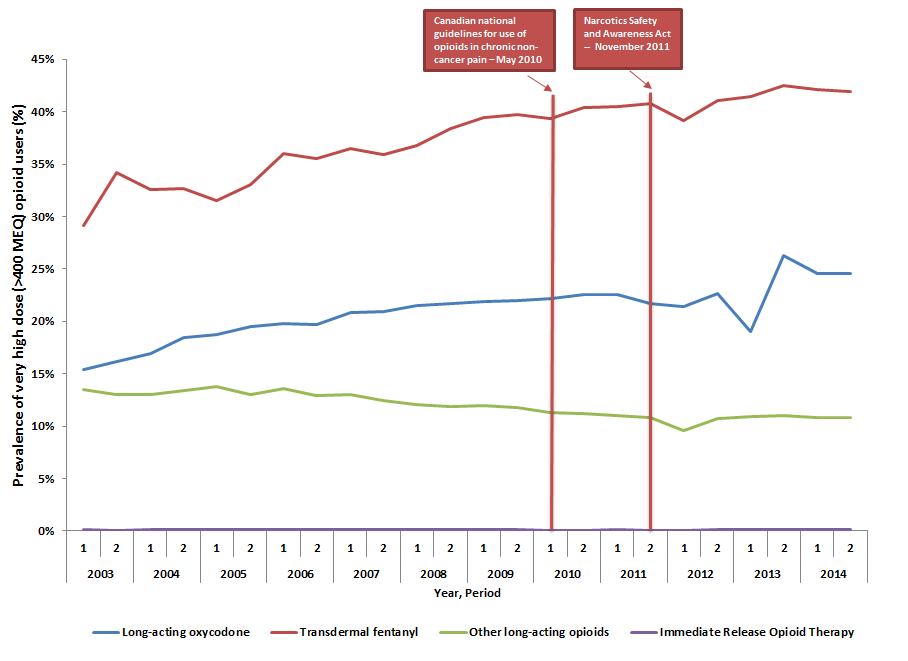

Supplement: S1 Fig — (PNG) [file pone.0167479.s001.png]

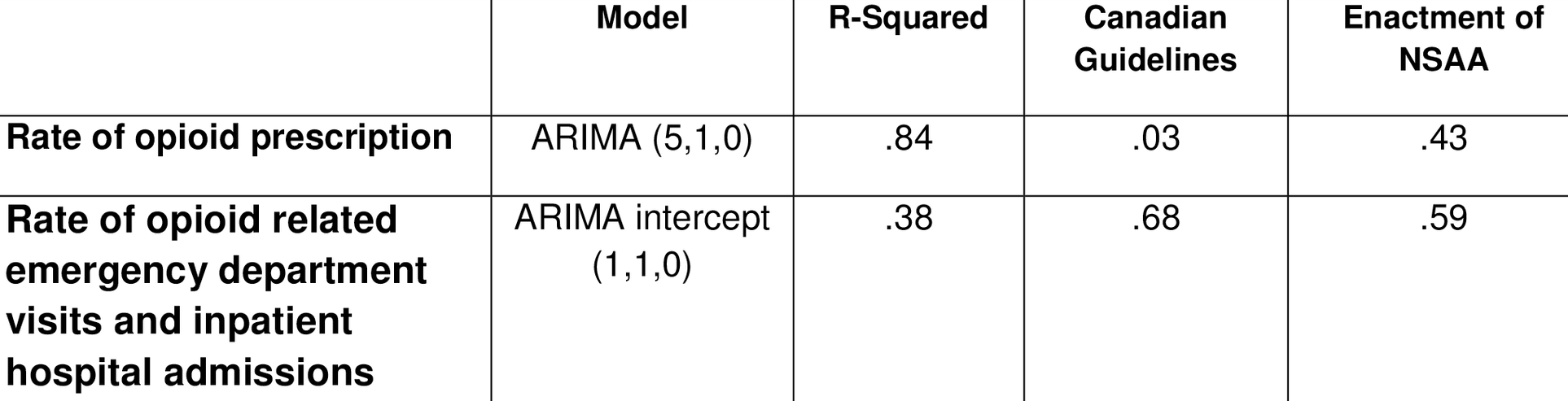

Supplement: S1 Table — (TIF) [file pone.0167479.s003.tif]
