# Supplementary material for: Correction: High-Dose Opioid Prescribing and Opioid-Related Hospitalization: A Population-Based Study
Source: PLoS One. 2017 Jan 20;12(1):e0170834. doi: 10.1371/journal.pone.0170834 (PMC5249127; doi:10.1371/journal.pone.0170834)
Supplement: S2 File — (PDF) [file pone.0170834.s002.pdf]

PLOS | ONE

RESEARCH ARTICLE

High-Dose Opioid Prescribing and Opioid-Related Hospitalization: A Population-Based Study

Luke Spooner[1], Kimberly Fernandes[2], Diana Martins[2], David Juurlink[2,3], Muhammad Mamdani[2,4,5,6,7], J. Michael Paterson[2,5,8], Samantha Singh[2], Tara Gomes[2,5,6,7]*

1 Faculty of Pharmaceutical Sciences, University of British Columbia, Vancouver, Canada, **2** Institute for Clinical Evaluative Sciences, Toronto, Ontario, Canada, **3** Sunnybrook Research Institute, Sunnybrook Health Sciences Centre, Toronto, Ontario, Canada, **4** Department of Medicine, St. Michael's Hospital, Toronto, Ontario, Canada, **5** Institute of Health Policy, Management, and Evaluation, University of Toronto, Toronto, Ontario, Canada, **6** Leslie Dan Faculty of Pharmacy, University of Toronto, Toronto, Ontario, Canada, **7** Li Ka Shing Knowledge Institute, St. Michael's Hospital, Toronto, Ontario, Canada, **8** Department of Family Medicine, McMaster University, Hamilton, Ontario, Canada

* GomesT@smh.ca

CrossMark
click for updates

OPEN ACCESS

Citation: Spooner L, Fernandes K, Martins D, Juurlink D, Mamdani M, Paterson JM, et al. (2016) High-Dose Opioid Prescribing and Opioid-Related Hospitalization: A Population-Based Study. PLoS ONE 11(12): e0167479. doi:10.1371/journal.pone.0167479

Editor: Dena L. Schanzer, Public Health Agency of Canada, CANADA

Received: April 4, 2016

Accepted: November 15, 2016

Published: December 14, 2016

Copyright: © 2016 Spooner et al. This is an open access article distributed under the terms of the Creative Commons Attribution License, which permits unrestricted use, distribution, and reproduction in any medium, provided the original author and source are credited.

Data Availability Statement: Data cannot be made publicly available due to the ethical and legal requirements implemented by the Institute for Clinical Evaluative Sciences and Ontario's Information and Privacy Commissioner (IPC). Public availability of ICES data would compromise patient privacy. More information can be found here: http://www.ices.on.ca/Data-and-Privacy/Privacy%20at%20ICES. Requests to access data may be submitted to Data & Analytic Services at das@ices.on.ca or via the following link http://www.ices.on.ca/Data-Services.Additional data.

Abstract

Aims

To examine the impact of national clinical practice guidelines and provincial drug policy interventions on prevalence of high-dose opioid prescribing and rates of hospitalization for opioid toxicity.

Design

Interventional time-series analysis.

Setting

Ontario, Canada, from 2003 to 2014.

Participants

Ontario Drug Benefit (ODB) beneficiaries aged 15 to 64 years from 2003 to 2014.

Interventions

Publication of Canadian clinical practice guidelines for use of opioids in chronic non-cancer pain (May 2010) and implementation of Ontario's Narcotics Safety and Awareness Act (NSAA; November 2011).

Measurements

Three outcomes were explored: the rate of opioid use among ODB beneficiaries, the prevalence of opioid prescriptions exceeding 200 mg and 400 mg morphine equivalents per day, and rates of opioid-related emergency department visits and hospital admissions.

Funding: This study was supported by a grant from the Ontario Ministry of Health and Long-Term Care (MOHLTC) Health System Research Fund. It also was supported by the Institute for Clinical Evaluative Sciences (ICES), an independent, non-profit research institute funded by an annual grant from the MOHLTC. The opinions, results, and conclusions reported in this article are those of the authors and are independent from the funding sources. No endorsement by ICES or the Ontario MOHLTC is intended or should be inferred. Parts of this material are based on data and information compiled and provided by the Canadian Institute for Health Information (CIHI). However, the analyses, conclusions, opinions and statements expressed herein are those of the authors and not necessarily those of CIHI. The Ontario MOHLTC provided support in the form of a research grant for MM, DJ, TG, but did not have any additional role in the study design, data collection and analysis, decision to publish, or preparation of the manuscript. The specific roles of these authors are articulated in the 'author contributions' section. Ms. Fernandes had full access to all of the data in the study and takes responsibility for the integrity of the data and the accuracy of the data analysis.

Competing Interests: Competing interests of the authors include: MM has served on advisory boards and/or received honorariums for unrelated work from Astra Zeneca, Bristol-Myers Squibb, Eli Lilly and Company, GlaxoSmithKline, Hoffman La Roche, Novartis, Novo Nordisk, and Pfizer; KF is currently employed at Hoffman La Roche, however during the conduct of the project KF had no involvement with Hoffman La Roche and no competing interests as this employment initiated after the completion of data analysis and interpretation. MP and TG report grants from the Ontario Ministry of Health and Long-Term Care during the conduct of the study; no other relationships or activities that could appear to have influenced the submitted work. This does not alter our adherence to PLOS ONE policies on sharing data and materials.

Findings

Over the 12 year study period, the rate of opioid use declined 15.2%, from 2764 to 2342 users per 10,000 ODB eligible persons. The rate of opioid use was significantly impacted by the Canadian clinical practice guidelines (p-value = .03) which led to a decline in use, but no impact was observed by the enactment of the NSAA (p-value = .43). Among opioid users, the prevalence of high-dose prescribing doubled (from 4.2% to 8.7%) over the study period. By 2014, 40.9% of recipients of long-acting opioids exceeded daily doses of 200 mg morphine or equivalent, including 55.8% of long-acting oxycodone users and 76.3% of transdermal fentanyl users. Moreover, in the last period, 18.7% of long-acting opioid users exceeded daily doses of 400 mg morphine or equivalent. Rates of opioid-related emergency department visits and hospital admissions increased 55.0% over the study period from 9.0 to 14.0 per 10,000 ODB beneficiaries from 2003 to 2013. This rate was not significantly impacted by the Canadian clinical practice guidelines (p-value = .68) or enactment of the NSAA (p-value = .59).

Conclusions

Although the Canadian clinical practice guidelines for use of opioids in chronic non-cancer pain led to a decline in opioid prescribing rates among ODB beneficiaries these guidelines and subsequent Ontario legislation did not result in a significant change in rates of opioid-related hospitalizations. Given the prevalence of high dose opioid prescribing in this population, this suggests that improved strategies and programs for the safe prescribing of long-acting opioids are needed.

Introduction

Long-term opioid treatment for non-cancer pain has become common, although little evidence supports the practice[1,2]. As a result, over the last two decades, significant increases in opioid prescribing rates and average prescription volumes have been documented in both the United States [3] and Canada [4]. These trends are concerning because high-dose opioid therapy is associated with considerable morbidity and mortality, including drug toxicity, overdose death, falls, fractures, and motor vehicle injury [5–8]. As a result, clinical practice guidelines have been developed in Canada and recently in the United States with the goal of promoting safe and effective prescribing of opioids for chronic pain [9,10]. Furthermore, policies have been implemented at regional levels, including Washington's Interagency Guideline on Prescribing Opioids for Pain[11], Florida's implementation of a prescription drug monitoring program[12], New York's implementation of an internet system for tracking over-prescribing [13], and Staten Island's targeted public health interventions to address its opioid mortality rates [14].

In Canada, interventions aimed at reducing opioid use have included the publication of national clinical practice guidelines regarding use of opioids in chronic non-cancer pain in May 2010[15] and the Ontario's Narcotics Safety and Awareness Act (NSAA) in November 2011 [16]. The publication of national clinical practice guidelines provided evidence-based recommendations on opioid indications, selection, precautions and monitoring to Canadian physicians with the focus of reducing opioid-related harms such as addiction and overdose [15].

Furthermore, these were the first national guidelines in Canada to establish dose thresholds for opioid prescribing in chronic non-cancer pain, and thus it is anticipated that this would lead to more prudent opioid dosing among physicians across Canada. Similarly, a key component of the NSAA was the requirement for prescriptions for all narcotics and other controlled substances dispensed in Ontario to be disclosed to the Ministry of Health and Long-Term Care for monitoring and surveillance. Therefore, it was anticipated that the clinical practice guidelines and enactment of this legislation would lead to more prudent opioid prescribing across Ontario that would lead to decreased opioid prescribing, thus reducing risks of opioid overdose.

Some evidence suggests that clinical practice guidelines and prescription drug monitoring programs have influenced opioid dose, diversion and related hospitalizations. For example, in Washington State, the 2007 opioid dosing guidelines were associated with declines in the average daily dose of long-acting opioids dispensed, as well as the proportion of individuals treated with doses exceeding 120 mg/day MEQ [17]. The implementation of a prescription drug monitoring program in Florida reduced opioid diversion rates and oxycodone-related mortality [18,19], while targeted interventions in Staten Island resulted in decreases in opioid prescriptions, high-dose opioid prescribing, and opioid-related mortality [14].

Research published in Canada found that rates of opioid prescribing increased by over 16% between 2003 and 2008, and that between 20% and 30% of long-acting opioid users were dispensed high dose therapy in 2008 [4]. However, it is not known whether the 2010 Canadian clinical practice guidelines or the enactment of Ontario's NSAA have influenced opioid prescribing or adverse events. Therefore, we assessed whether these policies and guidelines impacted opioid prescribing and opioid-related adverse outcomes in Ontario.

Methods

Setting

We conducted a time series intervention (interrupted) analysis in a cohort of individuals aged 15 to 64 eligible for drug coverage through the Ontario Public Drug Program (OPDP) between January 1st, 2003 and December 31st, 2014. Ontario is Canada's most populous province, with a population of 13.7 million in 2014. This study was approved by the research ethics board of Sunnybrook Health Sciences Centre in Toronto, Ontario, Canada.

Data Sources

We obtained opioid prescription data from the Ontario Drug Benefit (ODB) database, which contains information on all prescriptions dispensed to eligible Ontario residents. In Ontario, eligibility criteria for drug coverage in this demographic includes unemployment, disability, high prescription drug costs relative to net household income, receipt of home care services, and residence in a long-term care facility. We used the Canadian Institute for Health Information's Discharge Abstract Database (CIHI-DAD), and National Ambulatory Care Reporting System (CIHI-NACRS) to identify opioid-related hospital admissions and emergency department visits. We used the Ontario Cancer Registry to identify past cancer diagnoses and the Ontario Health Insurance Plan (OHIP) Claims Database to identify physician claims for palliative care services. These datasets are housed in a data repository at the Institute for Clinical Evaluative Sciences (ICES, www.ices.on.ca), are linked using unique, encoded identifiers based on patient health card numbers, and are regularly used for research purposes [4,6,7,20]. The data was analyzed anonymously at ICES, and was approved by the Research Ethics Board of Sunnybrook Health Sciences Centre, Toronto, Canada.

Rate of Opioid Use

Among the cohort of ODB eligible individuals aged 15 to 64 years, we identified all subjects who received at least one opioid prescription in each biannual period (January to June and July to December) over the study period. Opioids included in the analyses were oxycodone, transdermal fentanyl, morphine, meperidine, hydromorphone and codeine. We excluded prescriptions for parenteral and intranasal preparations of opioids, as well as methadone, which is almost exclusively used for individuals with a history of opioid misuse in Ontario. To limit our observations to individuals using opioids for chronic non-cancer pain, we excluded individuals with any past diagnosis of cancer and those receiving palliative care in the 180 days prior to the beginning of each biannual period. We classified each individual into one of four hierarchical, mutually exclusive groups based on opioid therapy received in each biannual period: 1) long-acting oxycodone (regardless of other opioid therapy), 2) transdermal fentanyl (with no long-acting oxycodone), 3) other long-acting opioids (with no long-acting oxycodone or fentanyl), and 4) immediate-release single agent opioids or immediate-release opioids in combination with acetaminophen or acetylsalicylic acid (with no long-acting opioid). We reported the number of opioid users per 10,000 ODB-eligible individuals by opioid therapy group for each biannual period over the study period. For each biannual period, ODB-eligible individuals were defined as all individuals who received at least one prescription in the biannual period for any drug covered by ODB excluding MedCheck or flu vaccine.

Prevalence of High-Dose Opioid Use

The prevalence of high-dose opioid prescribing was determined within the group of opioid recipients defined above. In each biannual period, each individual's average daily dose of opioid dispensed was calculated in morphine equivalents (MEQ) using ratios employed by the Canadian Guideline for Safe and Effective Use of Opioids for Chronic Non-Cancer Pain [21].

The average daily dose dispensed in each biannual period is based on average opioid use over a period of 90 days and was calculated as follows: We identified the first opioid prescription in the period, defining this as the index date. We then included all other prescriptions dispensed within the 100 days before or 90 days following the index date. For prescriptions with a days' supply overlapping the beginning or end of the 90 day period, or that overlapped with the end of the biannual period, the amount of opioid dispensed was adjusted accordingly to only include opioids prescribed for use within the 90 day period (Fig 1). The total volume dispensed was summed over the 90 day period, and the daily dose was calculated by dividing this volume by the shorter of 90 days or the number of days between index date and end of the biannual period. This is similar to the approach employed in previously published studies[4,7], but is more rigorous as it incorporates overlapping prescriptions in the calculation of daily dose and excludes excess doses that would have been used outside of the 90 day window. Two groups of individuals were identified based on their average daily opioid dose dispensed: high-dose users (>200mg MEQ daily) and very high-dose users (>400mg MEQ daily). To determine the percentage of individuals by opioid therapy group who were high (>200MEQ) and very high dose (>400MEQ), we divided the total number of individuals who were high and very high dose by the total number of individuals in each of these group.

Rate of Opioid-Related Emergency Department and Hospital Admissions

We identified all opioid-related emergency department visits and hospital admissions among individuals in our cohort between January 1, 2003 and December 31, 2013. Hospitalizations

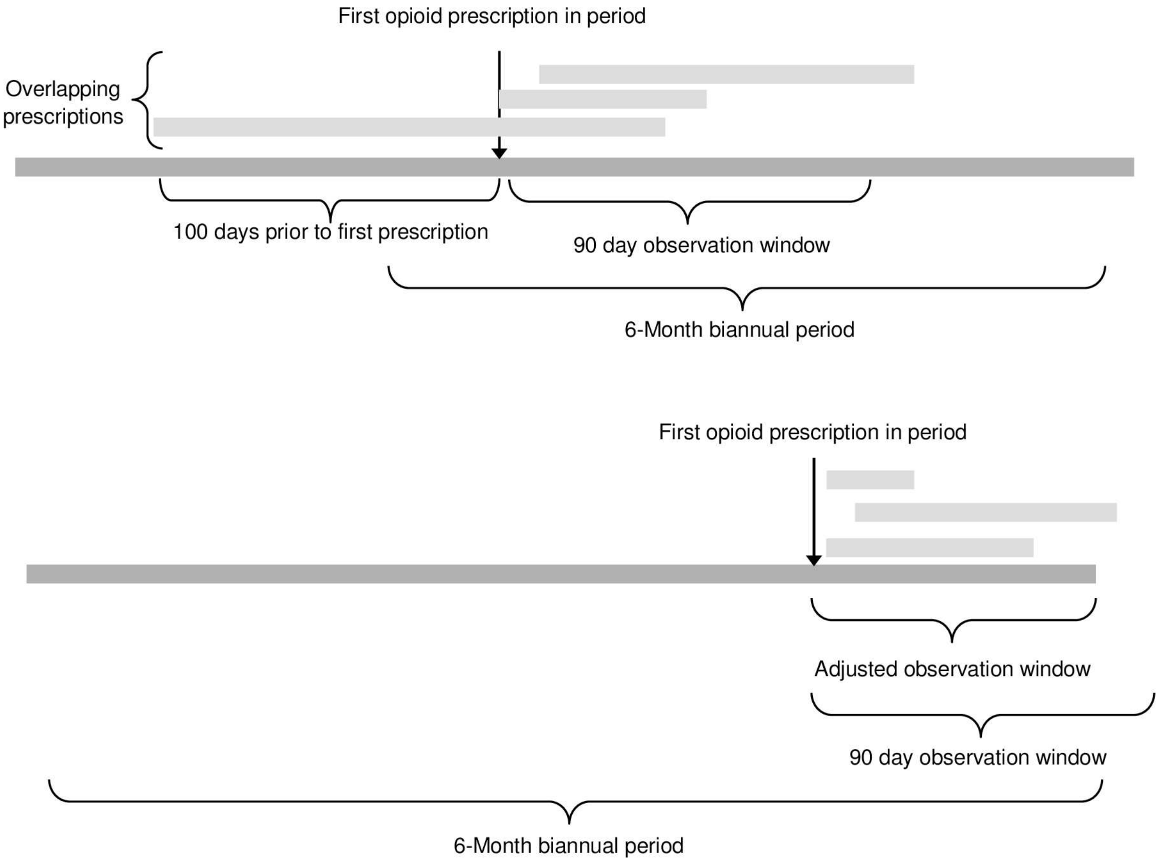

Fig 1. Study Design. Calculation of daily opioid dose in mg MEQ. A: All prescriptions dispensed within 90 days following the first opioid prescription including overlapping prescriptions from 100 days prior B: All prescriptions dispensed within either the 90 day period or between first prescription and end of the biannual period whichever was shorter. If the days supply overlapped the beginning or end of the 90 period or the end of the biannual period, the amount dispensed was adjusted to exclude excess drug.

doi:10.1371/journal.pone.0167479.g001

were identified using the CIHI-DAD and emergency department visits were identified using CIHI-NACRS. Opioid-related diagnoses were identified using International Classification of Disease, 10th revision (ICD-10) codes T40.0, T40.1, T40.2, T40.3, T40.4, and T40.6. If an individual visited an emergency department and was subsequently admitted, this was deemed a single visit.

Statistical Analysis

In this time series intervention (interrupted) analysis, we fit the biannual rate of opioid prescribing and rate of opioid-related hospitalizations with an autoregressive integrated moving average (ARIMA) model. We examined the impact of the introduction of the Canadian clinical practice guidelines (May 2010) and the NSAA (November 2011) on the time series data by including two ramp intervention functions in the model at the time points when the interventions were introduced (May 2010 and November 2011). A ramp function was used to detect a gradual change in the time series as this is the impact expected from the introductions of the policies. The t-statistic from the maximum likelihood estimation was used to determine if the ramp intervention function was a significant parameter in the ARIMA model. If the ramp function was a significant parameter (p-value$<=$ 0.05) in the ARIMA model it was deemed to have a significant impact on the time series. Intervention time series analysis is commonly

used to test the impact of an intervention on time series data, with the null hypothesis of no effect of the intervention on the time series of interest [22]. Stationarity and seasonality of the time series data was assessed using the 1. Augmented Dickey-Fuller unit root test 2.Autocorrelation plots and 3. Ljung-Box chi-square test for white noise. We assessed the autocorrelation, partial autocorrelation and inverse autocorrelation plots to identify model parameters. Final model specifications can be found in S1 Table. All analyses used a type 1 error rate of 0.05 as the threshold for statistical significance. The time series analysis was carried out using the SAS/ETS time Series Forecasting System. Analyses were carried out using SAS statistical software (v 9.3, EG 6.1; SAS Institute, Cary, NC).

Results

Over our 12-year study period, we identified 769,895 individuals who were dispensed at least one opioid prescription. Overall rates of opioid use remained relatively stable between 2003 and 2010 (Fig 2). The introduction of the Canadian clinical practice guidelines in May 2010 significantly impacted the rate of opioid use (p-value = .03) leading to a decline, from 2713 users per 10,000 ODB eligible persons in the first half of 2010 to 2342 users per 10,000 ODB eligible persons in the second half of 2014. The introduction of NSAA in November 2011 did not impact the rate of opioid use (p-value = .43).

Despite decreasing rates of opioid prescribing, the prevalence of high and very high opioid doses increased over our study period among those individuals who remained on opioid therapy. By the end of 2014, 40.9% of long-acting opioid users were treated with high daily doses (exceeding 200mg MEQ), and 18.7% were treated with very high daily doses (exceeding 400mg MEQ). Although the total number of long-acting oxycodone users declined considerably

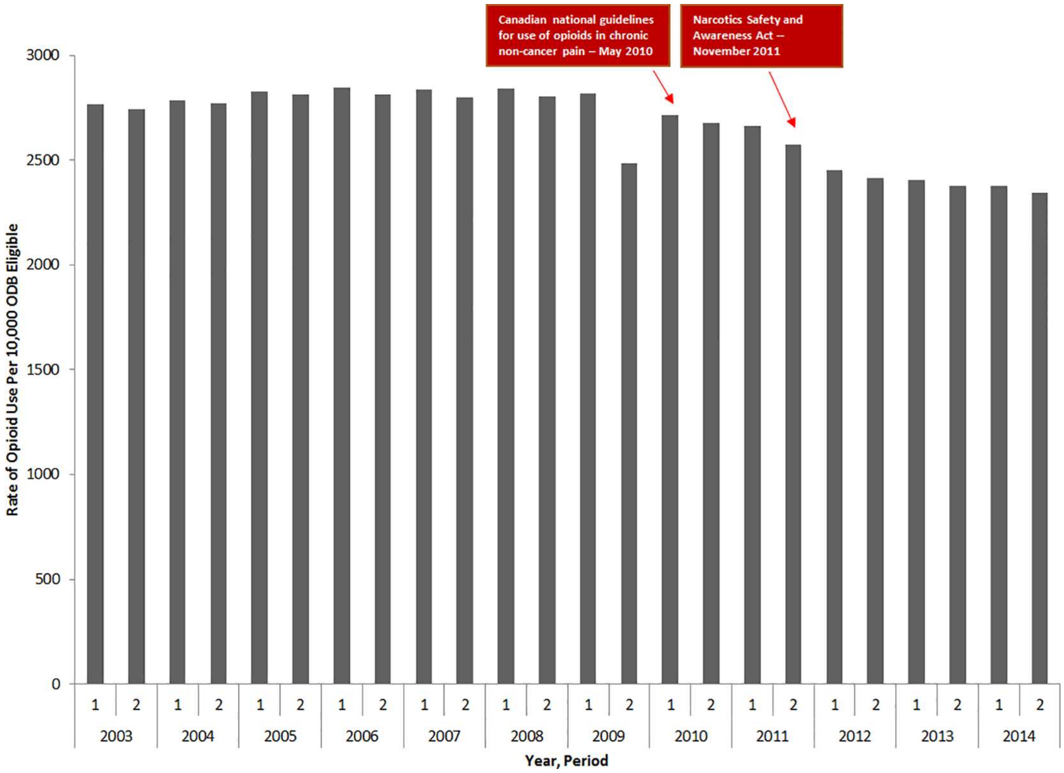

Fig 2. Rate of opioid users (per 10, 000 ODB eligible persons) between 2003 and 2014.

doi:10.1371/journal.pone.0167479.g002

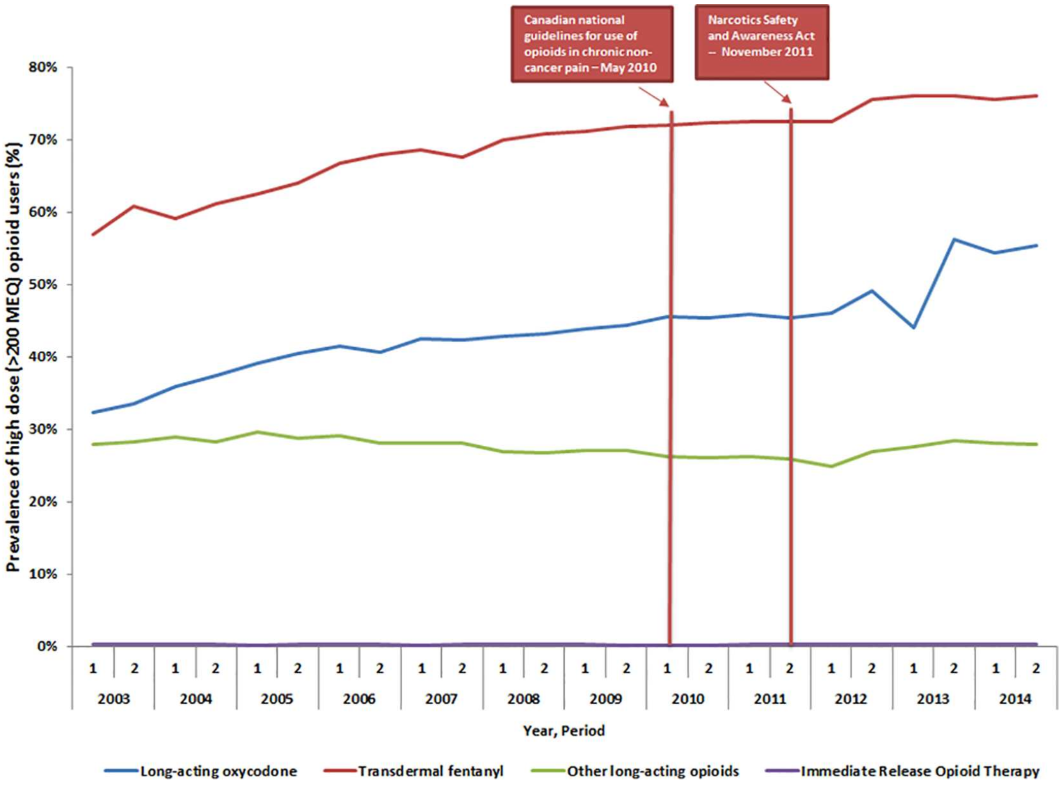

Fig 3. Percentage of opioid users considered high-dose users, by year and opioid group, between 2003 and 2014.

doi:10.1371/journal.pone.0167479.g003

following the move of this product to a prior authorization program on the public drug formulary (from 11,492 in second half of 2012 to 3,832 in the second half of 2013), the prevalence of high-dose use increased considerably among long-acting oxycodone users over this time (Fig 3). By 2014, 55.4% of long-acting oxycodone users were treated with high daily doses and 24.5% were treated with very high daily doses (Table 1). Following the restriction of access to long-acting oxycodone, considerable increases were evident in both the total number of fentanyl users (from 5,322 in the second half of 2012 to 6,193 in the second half of 2013) and other long-acting opioid users (from 15,668 in the second half of 2012 to 19,951 in the second half of 2013). Over this same period, the prevalence of high-dose fentanyl and other-long acting opioid use increased. In the second half of 2014, 76.1% of fentanyl users and 27.9% of other long-acting opioid recipients were treated with high doses (Fig 3). Additionally, 41.9% of fentanyl users and 10.8% of other long-acting opioid users exceeded very high dose thresholds by the end of the study period (S1 Fig).

Rates of opioid-related hospital visits increased 34.5%, from 9.0 per 10,000 ODB eligible persons in the first half of 2003 to 12.2 per 10,000 ODB eligible persons in the second half of 2004 (Fig 4), but remained relatively stable between 2005 and 2009. Between 2010 and 2013, rates increased again, rising 13.0% from 12.4 to 14.0 hospital visits per 10,000 ODB eligible persons. The rate of opioid-related hospitalizations was not significantly impacted by the Canadian clinical practice guidelines in May 2010 (p-value = .68) or the NSAA legislation in November 2011 (p-value = .59). In 2013, there were 1,621 opioid related hospital visits among public drug beneficiaries in Ontario.

Table 1. Summary of individuals who exceeded 200 and 400mg MEQ by period, overall, and stratified by opioid group.

Type and dose of Opioid	Jan-June 2003	Jan-June 2010	July-Dec 2011	July-Dec 2014
**Long-acting oxycodone**				
	**N = 4,501**	**N = 17,379**	**N = 17,774**	**N = 3,651**
Exceeded 200mg MEQ	1,455 (32.3%)	7,906 (45.5%)	8,051 (45.3%)	2,023 (55.4%)
Exceeded 400mg MEQ	691 (15.4%)	3,850 (22.2%)	3,855 (21.7%)	895 (24.5%)
**Transdermal fentanyl**				
	**N = 1,721**	**N = 4,207**	**N = 4,732**	**N = 6,049**
Exceeded 200mg MEQ	978 (56.8%)	3,027 (72.0%)	3,430 (72.5%)	4,605 (76.1%)
Exceeded 400mg MEQ	502 (29.2%)	1,657 (39.4%)	1,931 (40.8%)	2,533 (41.9%)
**Other long-acting opioids**				
	**N = 6,888**	**N = 11,747**	**N = 12,988**	**N = 20,528**
Exceeded 200mg MEQ	1,928 (28.0%)	3,088 (26.3%)	3,372 (26.0%)	5,723 (27.9%)
Exceeded 400mg MEQ	929 (13.5%)	1,332 (11.3%)	1,407 (10.8%)	2,220 (10.8%)
**Immediate-release single-agent and combination opioid therapy**				
	**N = 96,301**	**N = 116,068**	**N = 115,082**	**N = 115,415**
Exceeded 200mg MEQ	215 (0.2%)	218 (0.2%)	253 (0.2%)	362 (0.3%)
Exceeded 400mg MEQ	88 (0.1%)	102 (0.1%)	102 (0.1%)	111 (0.1%)
**Any type of opioid**				
	**N = 109,411**	**N = 149,401**	**N = 150,576**	**N = 145,643**
Exceeded 200mg MEQ	4,576 (4.2%)	14239 (9.5%)	15106 (10.0%)	12,713 (8.7%)
Exceeded 400mg MEQ	2,210 (2.0%)	6,941 (4.6%)	7,295 (4.8%)	5,759 (4.0%)

doi:10.1371/journal.pone.0167479.t001

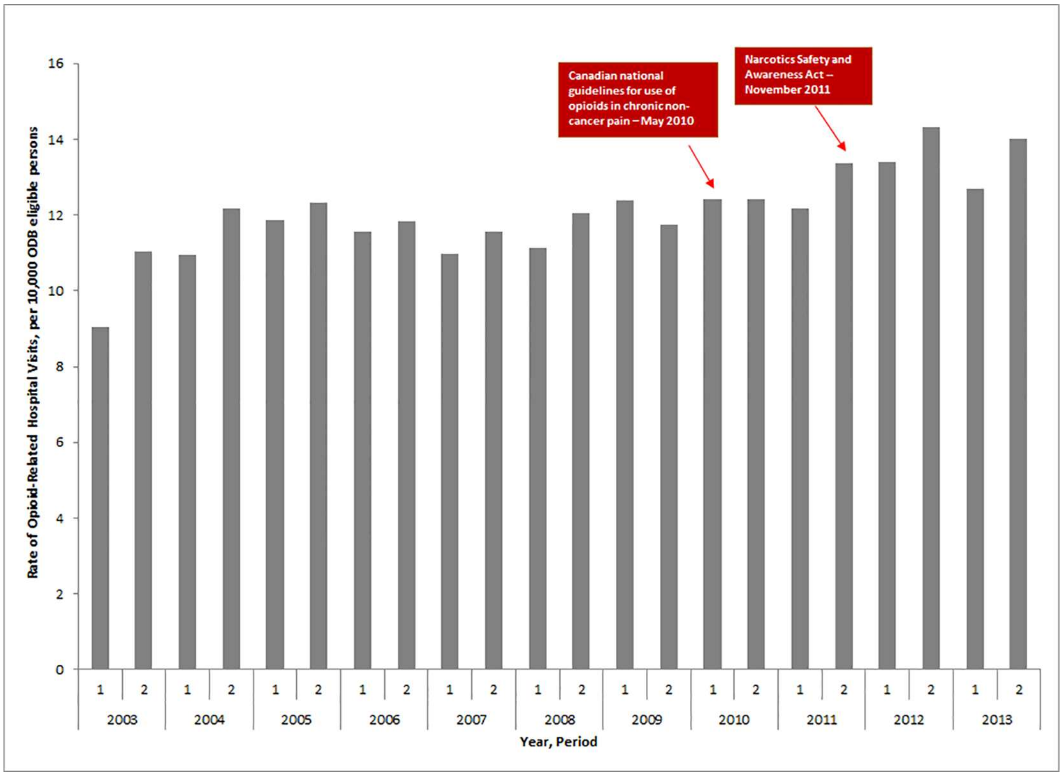

Fig 4. Rate of opioid related hospital or emergency room admissions (per 10,000 ODB eligible persons) between 2003 and 2013.

doi:10.1371/journal.pone.0167479.g004

Discussion

In this population-based study spanning 12 years, we found that the rate of opioid prescribing declined significantly in ODB beneficiaries following the introduction of the Canadian clinical practice guidelines in May 2010. This decline could be caused by the reduction of unnecessary opioid prescribing by physicians due to clearer indications for the use of opioids in the treatment. Additionally, this decline may be caused by a more comprehensive assessment of patient pain, medical, mental health and substance use history by physicians before initializing opioid therapy. However, over the same period, the prevalence of high-dose opioid prescribing increased among users of long-acting oxycodone and fentanyl who continued to access these drugs. Over half of long-acting oxycodone users and three-quarters of fentanyl users were dispensed more than 200 mg MEQ daily at the end of 2014. These shifts in high-dose opioid use may have impacted rates of hospital visits for opioid toxicity, which have also increased since 2010.

Trends in long-acting oxycodone dispensing are particularly interesting. We observed a large reduction in the number of ODB beneficiaries receiving this drug, but a corresponding increase in the prevalence of high-dose use (from 32.3% to 55.4% over the study period). This change is likely due to implementation of restrictions to the ODB program for coverage of a new tamper deterrent long-acting oxycodone product introduced by Purdue Pharma (OxyNeo®) in February 2012 that replaced the more widely available OxyContin®. At that time, the ODB program provided current long-acting oxycodone users one year to meet strict reimbursement criteria or transition to an alternative opioid, leading to a gradual reduction in the number of beneficiaries able to access long-acting oxycodone. Given the strict criteria required for ongoing access after this time (i.e. intolerance or failure on other long-acting opioids), individuals remaining on long-acting oxycodone after this period are more likely to be long-term opioid users, which is likely driving our findings of increased high-dose use. Furthermore, the higher number of users of other long-acting opioids, along with increases in the prevalence of high-dose opioid prescribing in these groups suggests that individuals previously treated with long-acting oxycodone switched to transdermal fentanyl and other long-acting opioids but continued to be prescribed high opioid doses.

Following the release of the Canadian clinical practice guidelines, rates of opioid use declined by 12% from 2010 to 2013, yet rates of opioid-related hospital visits increased 13% over this same period of time. Although we are unable to determine what led to increasing rates of opioid-related hospital visits, we hypothesize that this could be explained by increased illicit opioid use among individuals previously using prescription opioids or dosing errors as individuals were switched from oxycodone to alternative opioids with differing potency. For example, a study published in the United States reported that in a sample of patients using opioids for long-term opioid for non-cancer pain, 35% of long term opioid users met criteria for lifetime prescription opioid-use disorder [23]. Therefore, it is possible that those in our cohort who previously received prescription opioids may have transitioned to using illicit opioids upon restrictions imposed through the NSAA. This switch could have led to increased accidental opioid overdoses and resulting opioid-related hospital visits. Furthermore, once restrictions on long-acting oxycodone were introduced, many patients were required to transition to other prescription opioids reimbursed by the public drug program in Ontario. Therefore, given differing potency of long-acting oxycodone, there is potential for dosing errors that could lead to accidental opioid overdoses. Lastly, this increase may be related to the increasing prevalence of high-dose opioid use, which aligns with findings following the release to the Washington State guidelines [24], and suggests that guidelines and legislation alone may not be sufficient to reduce opioid-related toxicity, particularly in an environment of increasing prevalence of high-dose opioid use

Limitations

Several limitations of our study merit discussion. First, although all Ontarians have universal access to health care services, prescription drug coverage for those younger than 65 is generally restricted to the socioeconomically disadvantaged individuals, and therefore our findings may not be generalizable to other patients. Second, although we report opioid prescribing, we only have access to filled prescriptions from the ODB Program. Therefore our estimates represent the volume of opioids that are filled by the patients, and do not include information on prescriptions that were written, but never filled by a pharmacy. Third, calculated daily dose in mg MEQ was estimated from filled prescriptions from the ODB program and so unused prescription medications, and drugs obtained illicitly or paid for with cash were not identified. Therefore, our estimates of daily opioid dose may be underestimates of the true daily dose of opioids used by our cohort. Fourth, we used the Ontario Cancer Registry to exclude individuals with a past cancer diagnosis from our study cohort. Although this registry is reported to be over 95% complete, it is possible that we missed a small number of cancer diagnoses, thus mis-classifying these patients as opioid users for chronic non-cancer pain [25]. Finally, we were unable to determine if opioid-related emergency department visits and hospital admissions were a result of prescribed or non-prescribed opioids.

Conclusion and Implications

In summary, we found that although the Canadian clinical practice guidelines may have led to moderate reductions in opioid prescribing among Ontario Public Drug Program beneficiaries, these guidelines and subsequent enactment of the NSAA legislation have not led to significant changes on rates of opioid-related overdose. These findings provide insight as to the potential impact of both policies and guidelines in the area of opioid misuse and abuse and suggest that, while progress is being made in Canada, improved strategies and programs surrounding the prescribing of long-acting opioids–particularly at high doses—are needed.

Supporting Information

S1 Fig. Percentage of opioid users considered very high dose users, by year and opioid group, between 2003 and 2014
(PNG)

S2 Fig. Strengthening the Reporting of Observational Studies in Epidemiology (STROBE) Checklist
(PDF)

S1 Table. Details of the Time Series Analyses.
(TIF)

Author Contributions

Conceptualization: LS TG.

Data curation: KF.

Formal analysis: LS KF DM TG.

Funding acquisition: TG MM DJ.

Methodology: LS KF DM DJ MM JMP TG SS.

Project administration: SS.

Supervision: TG.

Visualization: LS TG.

Writing – original draft: LS TG.

Writing – review & editing: LS KF DM DJ MM JMP SS TG.

References

1. Boudreau D, Von Korff M, Rutter CM, Saunders K, Ray TG, Sullivan MD, et al. Trends in long-term opioid therapy for chronic non-cancer pain. Pharmacoepidemiol Drug Saf. 2009; 18:1166–1175. doi: 10.1002/pds.1833 PMID: 19718704
2. Chou R, Turner JA, Devine EB, Hansen RN, Sullivan SD, Blazina I et al. The Effectiveness and Risks of Long-Term Opioid Therapy for Chronic Pain: A Systematic Review for a National Institutes of Health Pathways to Prevention Workshop. Ann Intern Med. 2015; 162:276–286 doi: 10.7326/M14-2559 PMID: 25581257
3. Kenan K, Mack K, Paulozzi L. Trends in prescriptions for oxycodone and other commonly used opioids in the United States, 2000–2010. Open Med. 2012; 6:e41–47. PMID: 23696768
4. Gomes T, Juurlink DN, Dhalla IA, Mailis-Gagnon A, Paterson JM, Mamdani MM. Trends in opioid use and dosing among socio-economically disadvantaged patients. Open Med. 2011; 5:e13–22. PMID: 22046214
5. Bohnert AS, Valenstein M, Bair MJ, Ganoczy D, McCarthy JF, Ilge MA et al. Association between opioid prescribing patterns and opioid overdose-related deaths. JAMA. 2011; 305:1315–1321. doi: 10.1001/jama.2011.370 PMID: 21467284
6. Gomes T, Redelmeir DA, Juulink DN, Dhaooa IA, Camacho X, Mamdani MM. Opioid dose and risk of road trauma in Canada: a population-based study. JAMA Intern Med. 2013; 173:196–201. doi: 10.1001/2013.jamainternmed.733 PMID: 23318919
7. Gomes T, Mamdani MM, Dhalla IA, Paterson JM, Mamdani MM. Opioid dose and drug-related mortality in patients with nonmalignant pain. Arch Intern Med. 2011; 171:686–691. doi: 10.1001/archinternmed.2011.117 PMID: 21482846
8. Saunders KW, Dunn KM, Merrill JO, Sullivan M, Weisner C, Braden JB et al. Relationship of Opioid Use and Dosage Levels to Fractures in Older Chronic Pain Patients. J Intern Med. 2010; 25:310–315.
9. Chou R, Fanciullo GJ, Fine PG, Adler JA, Ballantyne JC, Davies P et al. Clinical guidelines for the use of chronic opioid therapy in chronic noncancer pain. J Pain. 2009; 10:113–130. doi: 10.1016/j.jpain.2008.10.008 PMID: 19187889
10. Dowell D, Haegerich TM, Chou R. CDC Guideline for Prescribing Opioids for Chronic Pain—United States, 2016. JAMA. 2016; 315(15):1624–1645. doi: 10.1001/jama.2016.1464 PMID: 26977696
11. Washington State Agency Medical Directors' Group. Interagency guideline on opioid dosing for chronic non-cancer pain: an educational pilot to improve care and safety with opioid treatment [Internet]. Washington State Agency Medical Directors' Group; [cited 2016 Mar 3]. Available from: http://www.agencymeddirectors.wa.gov/files/opioidgdline.pdf
12. Florida Department of Health [Internet]. Welcome to E-FORCSE: the state of Florida's prescription drug monitoring program. Florida Department of Health [cited 2016 Mar 3]. Available from: http://www.floridahealth.gov/statistics-and-data/e-forcse/.
13. New York State Department of Health [Internet]. I-STOP/PMP—Internet System for Tracking Over-Prescribing—Prescription Monitoring Program. New York State Department of Health [cited 2016 Mar 3]. Available from: https://www.health.ny.gov/professionals/narcotic/prescription_monitoring/.
14. Paone D. Decrease in rate of opioid analgesic overdose deaths—Staten Island, New York City, 2011–2013. MMWR Morb Mortal Wkly Rep. 2015; 64:491–494. PMID: 25974633
15. Kahan M, Mailis-Gagnon A, Wilson L, Srivastava A. Canadian guideline for safe and effective use of opioids for chronic noncancer pain: clinical summary for family physicians. Part 1: general population. Can Fam Physician. 2011; 57:1257–1266, e1407-1218. PMID: 22084455
16. The Narcotics Safety and Awareness Act [statue on the Internet]. 2010 [cited 2016 Mar 3] Available from: www.health.gov.on.ca/en/public/programs/drugs/ons/ons_legislation.aspx.
17. Franklin GM, Mai J, Turner J, Sullivan M, Wickizer T, Fulton-Kehoe D. Bending the prescription opioid dosing and mortality curves: impact of the Washington State opioid dosing guideline. Am J Ind Med. 2012; 55:325–331. doi: 10.1002/ajim.21998 PMID: 22213274

18. Surratt HL, O'Grady C, Kurtz SP, Stivers Y, Cicero TJ, Dart RC, Chen M. Reductions in prescription opioid diversion following recent legislative interventions in Florida. Pharmacoepidemiol Drug Saf. 2014; 23:314–320. doi: 10.1002/pds.3553 PMID: 24677496

19. Delcher C, Wagenaar AC, Goldberger BA, Cook RL, Maldonado-Molina MM. Abrupt decline in oxycodone-caused mortality after implementation of Florida's Prescription Drug Monitoring Program. Drug Alcohol Depend. 2015; 150:63–68. doi: 10.1016/j.drugalcdep.2015.02.010 PMID: 25746236

20. Barbera M, Seow H, Husain A, Howell D, Atzema C, Sutradhar R, et al. Opioid prescription after pain assessment: a population-based cohort of elderly patient's with cancer. J Clin Oncol. 2012; 30:1095–1099. doi: 10.1200/JCO.2011.37.3068 PMID: 22370317

21. National Opioid Use Guideline Group (NOUGG). Canadian guideline for safe and effective use of opioids for chronic non-cancer pain [Internet]. Hamlton (Canada); c2010. [updated 2010 April 30; cited 2016 Mar 3] Available from: http://nationalpaincentre.mcmaster.ca/documents/opioid_guideline_part_b_v5_6.pdf

22. Gilmour S, Degenhardt L, Hall W, Day C. Using intervention time series analyses to assess the effects of imperfectly identifiable natural events: a general method and example. BMC Med Res Methodol. 2006; 6:16. doi: 10.1186/1471-2288-6-16 PMID: 16579864

23. Boscarino JA, Rukstalis MR, Hoffman SN, Han JJ, Erlich PM, Ross S et al. Prevalence of Prescription Opioid-Use Disorder Among Chronic Pain Patients: Comparison of the DSM-5 vs. DSM-4 Diagnostic Criteria. J Addict Dis. 2011; 30:185–94. doi: 10.1080/10550887.2011.581961 PMID: 21745041

24. Fulton-Kehoe D, Garg RK, Turner JA, Bauer AM, Sullivan MD, Wickizer TM et al. Opioid poisonings and opioid adverse effects in workers in Washington state. Am J Ind Med. 2013; 56:1452–1462. doi: 10.1002/ajim.22266 PMID: 24122929

25. Clark EA, Marrett LD, Kreiger N. Cancer registration in Ontario: a computer approach. IARC Sci Publ. 1991:246–257. PMID: 1894327
